# Cardiotoxicity in Biological Agent-Targeted Therapy for Rheumatoid Arthritis: ADR Signal Mining and Analysis of Food and Drug Administration Adverse Event Reporting System Database

**DOI:** 10.3389/fped.2021.716648

**Published:** 2021-10-12

**Authors:** Xiaoyan Tang, Xiaolin Xu, Ji Li, Bin Zhao

**Affiliations:** ^1^Department of Pediatrics, Peking Union Medical College Hospital (CAMS), Beijing, China; ^2^Department of Pharmacy, Beijing Children's Hospital, National Center for Children's Health, Capital Medical University, Beijing, China; ^3^Department of Pharmacy, Peking Union Medical College Hospital (CAMS), Beijing, China

**Keywords:** biologics, pharmacoepidemiology, rheumatoid arthritis, adverse, drug-induced cardiotoxicity

## Abstract

**Purpose:** Biologic agent-induced cardiotoxicity is markedly concerning. Rheumatoid arthritis (RA) treated with biologic agents is known to have the potential for cardiotoxicity; however, existing clinical evidence is not adequate to explain real-world patterns of cardiotoxicity. In this study, we quantify the risk of cardiotoxicity in patients with rheumatoid arthritis treated with biological agents.

**Methods:** Cardiotoxicity reports induced by four types of biologic agents, abatacept, adalimumab, tocilizumab, and etanercept were used to mine data from the FDA's adverse event reporting system (FAERS) database from January 1, 2004 through September 30, 2020. Reports of cardiotoxic events were analyzed using a reporting odds ratio (ROR) algorithm, the proportional reporting ratio (PRR), the Bayesian confidence propagation neural network (BCPNN), the multi-item gamma Poisson shrinker (MGPS), and logistic regression methods. We use the preferred term of the Medical Dictionary of Regulatory Activities to identify such events.

**Results:** A total of 3,969 reports of cardiotoxic events were identified involving biologic agents used for RA as the suspect drugs in this study, 317 reports of abatacept, 2,137 reports of adalimumab, 273 reports of tocilizumab, and 1,242 reports of etanercept. Adalimumab was the most reported, followed by etanercept. The proportion of death and disability outcomes reported for each targeted treatment represents approximately 20–25% of the total reported severe adverse events. In addition, relatively low cardiotoxicity reporting rates were found with abatacept.

**Conclusion:** Analysis of FAERS data offers a more precise profile on the characteristics and occurrences of cardiotoxic events. The findings are a clinical reminder to physicians that an increased vigilance concerning the cardiotoxic effects of biological agents needs to be implemented. Also, more comparative studies are required in the future to explain the mechanisms that cause these cardiac phenomena.

## Introduction

The advancements in molecular biology, immunological science, and drug research since the late 1990s have led to a variety of new treatment methods for rheumatoid arthritis (RA) and other systemic inflammatory diseases associated with autoimmunity (1). The major biological approaches in clinical use, including both medications made by molecular biologic techniques and small molecule kinase inhibitors, include agents that interfere with cytokine function, signal transduction, production, restrain the “second signal” required for T-cell activation, or exhaust B cells (2).

Biologic agents provide a targeted strategy in contrast to non-specific immunosuppressors traditionally used to the treatment of most inflammatory diseases. Cardiovascular disease (CVD) in autoimmune disease populations is increased, while systemic information is not only a risk factor but also a mechanism to initiate the development of CVD, such as cardiotoxicity (3). Atherosclerotic plaques prone to rupture are characterized by a thin fibrous cap, large lipid core, and significant inflammatory cell influx. Rupture of the intimal fibrous cap leads to thrombus propagation and may cause sudden vessel occlusion, resulting in myocardial infarction (MI) when the event occurs within a coronary artery. Cardiac ischemia triggers a complex inflammatory process leading to myocyte apoptosis, cardiac remodeling, and varying degrees of local myocardial dysfunction. Biological agent-induced cardiotoxicity may not only affect RA patients with existing cardiovascular complications but also affect patients with no known cardiac issues. In addition, targeted biological agent use might be associated with heart failure (HF). Thus, concerns about the possibility of adverse effects stem from randomized clinical trials of biological agents as a potential therapy for RA and from the FDA's early post-marketing surveillance data (4–6).

Patients with RA may be exposed to these agents for extended periods of time, as treatment is usually used to alleviate chronic symptoms. However, treatment may continue until the side effects become greater than the initial benefits of the therapy (7). In one study, cardiotoxicity may eventually lead to severe morbidity, causing premature death. Large clinical trials have shown cardiotoxicity as one of TNF inhibitors' most severe and significant adverse events of a biological agent (8).

Despite concerns, the risk of HF remains uncertain, and reassuringly, other studies have suggested that patients with RA receiving biological agents have an overall decrease in the incidence of cardiovascular events compared with those receiving nonbiological DMARD therapies (9). Recent clinical trials and population-based studies have suggested growing concern for cardiotoxicity with these targeted agents. Data regarding the risk of HF with the use of biological agents at the FDA-approved doses are inconclusive. Large epidemiologic studies explicitly examining the risk of cardiotoxicity are also lacking.

We sought to examine whether four biological agents for rheumatoid arthritis may increase the risk of cardiotoxicity by using a disproportionality analysis of the United States Food and Drug Administration (FDA) adverse drug reaction data (10).

Disproportionality analysis is a post-marketing data analysis that detects and quantifies new or partially known adverse drug events (11). It is used by the FDA to detect adverse drug reactions and has been used in previous publications to indicate associations between drugs and diseases (12).

## Materials and Methods

### Data Source

Data from the first quarter of 2004 to the third quarter of 2020 were retrieved from the FAERS database. FAERS is a voluntary, spontaneous reporting database containing information about adverse drug events and drug error reports submitted to the US FDA by healthcare professionals, patients, and manufacturers not only domestically but also from other regions. The FAERS database encodes adverse drug events (adverse drug event, ADE) using the preferred term (preferred term, PT) of the International Dictionary of Medical Languages (ICH) with high ADE original report normalized, the data structured, and with a large amount of information available (13).

FAERS data files describe demographic and administrative information (DEMO), drug information (DRUG), coded for adverse events (REAC), patient outcomes (OUTC), source of the reports (RPSR), therapy start dates and end dates of drug therapy (THER), as well as indications for use (INDI). First, we screened 5,660 reports from the FAERS database and removed duplicate records according to the FDA's recommendations with the same CASEID and FDA_DT. Then we selected the latest FDA_DT. Finally, 3,669 reports were included for further analysis.

### Adverse Events and Drug Identification

Cardiotoxicity was defined as cardiomyopathy and heart failure, as defined by the Cardiac Review and Evaluation Committee (CRCE). We investigated the REAC files for the comprehensive Medical Dictionary for Regulatory Activities (MedDRA) (14) v22.1 preferred terms (PTs) noted in the Supplementary Material related to cardiotoxicity adverse effects as follows: heart failure, fail cardiac, insufficient cardiac, cardiac insufficiency, cardiac failure, and weak heart, as defined by the Cardiac Review and Evaluation Committee (CRCE). Reports involving four kinds of biological agents for rheumatoid arthritis (RA) treatment (including abatacept, adalimumab, tocilizumab, and etanercept) were identified using text string searches for each drug by brand and generic names utilizing the www.drugbank.ca as a dictionary during the data mining process. We excluded qualified drugs listed as interacting or accompanying drugs, as well as those that did not have rheumatoid arthritis-related indications.

### Data Mining

Based on the rationale of Bayesian and disproportionality analysis, we employed the reported odds ratio (ROR), the proportional reporting ratio (PRR), the Bayesian confidence propagation neural network (BCPNN), and the multi-item gamma Poisson shrinker (MGPS) algorithms to study the association between drugs and a given adverse event. The equations and criteria for the four algorithms are listed in Table 1 (15).

**Table 1:** Summary of major algorithms used for signal detection.

**Algorithms**	Equation*	**Criteria**
ROR	ROR = (ab)(c/d)	95% CI > 1, N ≥ 2
	95%CI = e^ln(ROR) ± 1.96(1/a + 1/b + 1/c + 1/d)^0.5^^	
PRR	PRR = a(a + c)[b(b + d)]	PRR ≥ 2, χ2 ≥ 4, N ≥ 3
	χ^2^ = Σ[(O – E)2/E]; [O = a, E = (a + b)(a + c)/(a + b + c + d)]	
BCPNN	IC = log2a(a + b + c + d)/[(a + c)(a + b)]	IC025 > 0
	IC025 = e^ln(IC) −1.96(1/*a* + 1/*b* + 1/*c* + 1/*d*)^0.5^^	
MGPS	EBGM = a(a + b + c + d)/[(a + c)(a + b)]	EBGM05 > 2, N > 0
	EBGM05 = e^ln(EBGM) −1.64(1/*a* + 1/*b* + 1/*c* + 1/*d*)^0.5^^	

*ROR, reporting odds ratio; CI, confidence interval; N, the number of co-occurrences; PRR, proportional reporting ratio; χ^2^, chi-squared; BCPNN, Bayesian confidence propagation neural network; IC, information component; IC025, the lower limit of the 95% two-sided CI of the IC; MGPS, multi-item gamma Poisson shrinker; EBGM, empirical Bayesian geometric mean; EBGM05, the lower 90% one-sided CI of EBGM (10–17)*.

The number of reports of an event of interest associated with the drug of interest was compared with the number of reports of all other adverse events associated with this drug and the number of reports of this event associated with all other drugs listed in the database. The equation used for disproportionality analysis is shown in Table 2.

**Table 2:** Reporting odds ratio (ROR) algorithm list.

	**Drug of interest**	**All other drugs in the database**	**Total**
Adverse drug reaction of interest	A	B	A+B
All other adverse drug reaction	C	D	C+D
Total	A+C	B+D	A+B+C+D

*A, number of reports containing both the suspect drug and the suspect adverse drug reaction. B, number of reports containing the suspect adverse drug reaction with other medications (except the drug of interest). C, number of reports containing the suspect drug with other adverse drug reactions (except the event of interest). D, number of reports containing other medications and other adverse drug reactions*.

The outcome of patients with cardiotoxic events was extracted from the OUTC files. Outcome events were divided into three groups: disability (i.e., disability or congenital anomaly), death, and other serious outcomes (i.e., life-threatening, hospitalization, required intervention to prevent harm, and other outcomes). Events were classified from the most serious and/or specific groups of these mutually exclusive groups to prevent double counting.

### Statistical Analysis

We used descriptive analysis to summarize the clinical characteristics of the patients with cardiotoxicity caused by biological agents from the FAERS database. Then we checked the frequency distribution of the cardiotoxic events as well as the trends in health outcomes for each target treatment group. Furthermore, a disproportionate analysis with a reporting ratio (ROR) was used to evaluate the size of the event signal in the FAERS. The ROR was calculated using a case/non-case method. Specifically, case reports of cardiotoxic events and non-cases were all reports of adverse events other than cardiotoxicity. ROR estimated the probability of cardiotoxicity of patients exposed to each target drug in interest divided by those not exposed at all (all other drugs in the database). Information component (information component, IC) values were calculated based on the classical four-grid table (Table 1). If IC > 0, some connection between the drug and ADE is suspected.

Significant disproportionality, or in other words a possible signal, was defined as the lower bound of the 95% confidence interval (95% CI) exceeding 1. All statistical analyses used SAS descriptive analyses to summarize the characteristics of ADR for RA.

Report reporting regions and the time to onset of the biological agent-associated cardiotoxicity were compared using non-parametric tests, fatality rates, and reporters, and were compared using a Pearson's chi-square or Fisher exact tests. Statistical significance in this study was set at *p* < 0.05. All reported *p*-values were two-sided. Statistical analysis was performed using SPSS22.0.

## Results

### Descriptive Analysis

In the FAERS database, there are 10,271 adverse events related to biological agents and kinase inhibitors. The number of cardiotoxic events is 124,983, and among them, 3,936 events reports were identified as caused by biological agents and kinase inhibitors between January 2004 and September 2020. Table 3 provides the characteristics of the cardiotoxicity report for the above-targeted therapies.

**Table 3:** Clinical characteristics of patients with four agents associated with cardiotoxic events collected from the Food and Drug Administration's Adverse Event Reporting System (FAERS) database.

**Characteristics**	**Reports, *N* (%)**
OverallTotal reports related to cardiotoxicity	3,969
**Targeted therapy as suspect drug**	
Abatacept	317 (7.99)
Adalimumab	2,137 (53.84)
Tocilizumab	273 (5.91)
Etanercept	1,242 (31.29)
**Reporting region**	
North America	1,932 (48.68)
Europe	566 (14.26)
Asian	192 (4.83)
South America	148 (3.72)
Oceania	23 (0.58)
Africa	37 (0.93)
Unknown or missing	1,121 (28.24)
**Reporters**	
Health-care professional	2,237 (56.36)
Non-health-care professional	1,563 (38.70)
Unknown or missing	169 (4.26)
**Patient sex**	
Male	1,358 (34.21)
Female	2,450 (61.72)
Unknown or missing	161 (4.06)
**Patient age group (years)**	
<18	26 (0.66)
18–44	176 (4.43)
45–64	960 (24.19)
65–74	813 (20.48)
75–84	548 (13.80)
>85	110 (2.78)
Unknown or missing	1,315 (33.13)
**Report-related health outcomes**	
Death	932 (23.48)
Disability	92 (2.31)
Hospitalization	2,468 (62.18)
Life-threatening	242 (6.10)
Required intervention	21 (0.52)

*FAERS, Food and drug administration's adverse event reporting system*.

The highest number of cardiotoxic cases was adalimumab (n½), followed by etanercept. The patients were primarily female (61.72%), rather than male (34.21%). The most common age groups were 45–64 years (24.19%), with an average female age of 50.64 ± 12.73 years and 60.18 ± 11.78 years for males. Many of the cases originated from North America. Approximately 56.3% of the reports were submitted by healthcare professionals (i.e., periodic and expedited). The proportions of reports of severe events with a deadly outcome was 23.4% across all agents, while the disability outcome represented 2.31% across all agents. According to the data collected, most patients were hospitalized.

### Disproportionality Analysis and Bayesian Analysis

Overall, cardiotoxic event signals were detected by the criteria of four algorithms. The results are listed in Table 4. Tocilizumab showed higher ROR and PRR, but had no significant relationship with cardiotoxic events. Abatacept was also markedly associated with cardiotoxicity [ROR = 0.69, 95% CI (0.61–0.77)]. For adalimumab, the analysis of FAERS data suggests a significant association with cardiotoxicity [ROR = 0.48 95% CI (0.46–0.50)].

**Table 4:** Associations of different single drugs with cardiotoxic events.

**Drug name**	**N**	**ROR(95% two-sided CI)**	**PRR (χ^2^)**	**IC(95% one-sided CI)**	**EBGM (95% one-sided CI)**
Abatacep	317	0.69 (0.61,0.77)	0.69 (41.37)	–	0.69 (0.63)
Adalimumab	2,137	0.48 (0.46,0.50)	0.49 (1,120.91)	–	0.49 (0.48)
Tocilizumab	273	0.94 (0.82,1.05)	0.94 (1.13)	–	0.93 (0.84)
Etanercept	1,242	0.26 (0.24,0.27)	0.26 (2,518.11)	–	0.27 (0.26)
All agents combined	3,969				1.314 (1.05–1.65)

*ROR, reporting odds ratio; CI, confifidence interval; PRR, proportional reporting ratio; χ^2^, chi-squared; IC, information component; EBGM, empirical Bayes geometric mean*.

### Trends Over Time in Cardiotoxicity Event Reporting

**Figure 1:**
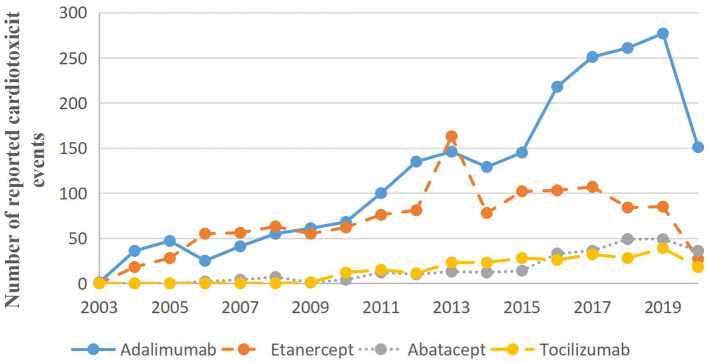
Cardiotoxicity reports associated with targeted therapy for rheumatoid arthritis by year.

Figure 1 illustrates the reporting of cardiotoxicity events with targeted therapies over time. The cardiotoxicity reports of adalimumab remained elevated over time. However, careful illustration is needed about the trends, as the reporting rates they reflect are not adjusted for usage. Adalimumab may be different from the use patterns of other drugs as it has been on the market longer.

### Number of Report-Related Health Outcomes

**Figure 2:**
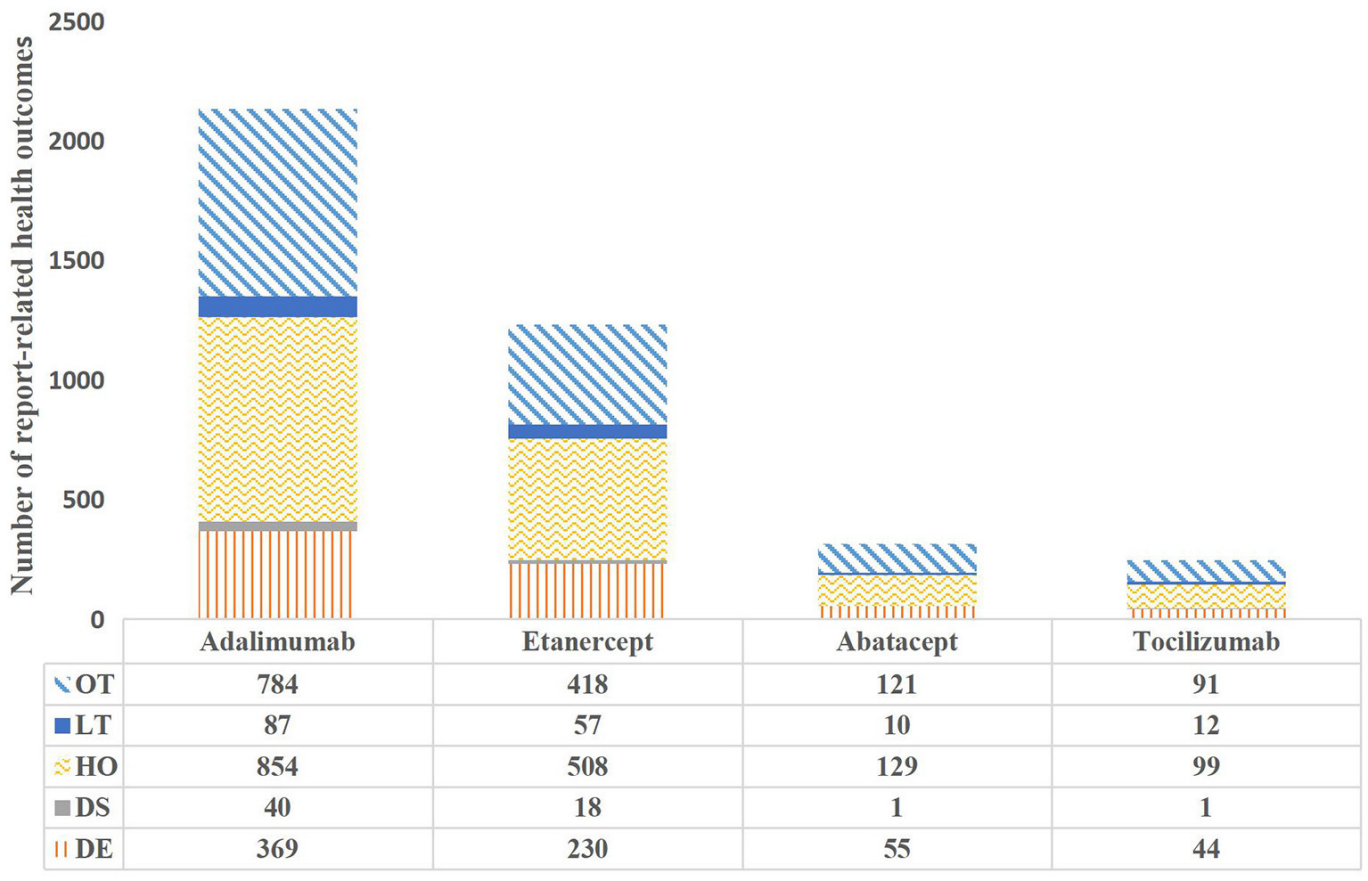
Health outcomes in reports involving cardiotoxicity with the targeted therapies.

Figure 2 shows that the proportion of serious events reported with death results is 16.11–18.51%, while the disability results account for 2.25–3.17% reported by all the agents. Thus, the results showed that the proportion of death or disability reported in etanercept was higher than the other biological agents.

## Discussion

The large publicly available adverse reaction database—FAERS database was used to mine and analyze the ADE signals of the four biological agent using Bayesian and disproportionality analysis. This method is a commonly used signal mining method, divided into frequency number method (ROR and PRR) and Bayesian (e.g., BCPNN and MGPS). Clinical trials of the four drugs did not focus on cardiovascular heart failure. After consulting the drug insert released by the US FDA, it was found that neither tocilizumab nor abatacept mentioned abnormal cardiotoxicity. Only adamuzumab and etanercept inserts stated, “It may aggravate the condition.”

Much of the early data on biologics and cardiovascular disease were derived from their use in rheumatologic populations. Moreover, biologic therapies have experienced a marked expansion as a therapeutic option for cardiovascular disease (CVD) (16). Furthermore, other studies have shown TNF-a to confer cardiomyocyte resistance to ischemic injury through a mechanism similar to preconditioning (17). When given before myocardial ischemia, low doses of TNF-a seem to improve myocyte survival, whereas higher doses of TNF-a increase the size of infarction. Long-term safety monitoring has shown a low incidence of cardiovascular events associated with tocilizumab (18, 19). Prescriber vigilance has also increased after recognition of adverse cardiovascular effects, particularly HF.

The FDA published a summary of 47 cases of HF associated with etanercept to the Adverse Events Response System (AERS) through February 2002 (20). Thirty-eight of these patients developed new-onset HF, and 9 experienced an HF exacerbation. Among the 38 patients with new-onset HF, 19 had no identifiable risk factors such as coronary artery disease, hypertension, history of myocardial infarction, or diabetes, and 10 were under the age of 50. A lower HF event rate (0.05 per 100 patient-years) was noted in an analysis of adalimumab's the United States post-marketing safety with 55,384 patient-years of exposure (21). Reports of cardiotoxicity by tocilizumab and abatacept are rare. One case reported a severe ADR and cardiotoxicity during the first infusion of tocilizumab injection.

ADE signals induced by cardiotoxicity of four biological agents (etanercept, adalimumab, abatacept, and tocilizumab) were analyzed. Other drugs used for the treatment of RA were attempted to be analyzed, but because of the small number, they cannot be computed with the ROR algorithm. Suspicious signals were found within the ROR, PRR, and BCPNN signals of the three drugs, abatacept, adalimumab, and etanercept. Moreover, the BCPNN method was relatively highly stable, no positive signal was detected by IC. This suggests a risk of inducing cardiotoxicity. The incidence of the four biological agents were adalimumab 53.84%, etanercept 31.29%, abatacept 7.99%, and tocilizumab 5.91%. The incidence of “Death” of the four agents were adalimumab 17.26%, etanercept 18.5%, abatacept 17.35%, and tocilizumab 16.1%. This suggests that the biological agents causes severe cardiac events, poor prognosis, and high mortality. One study showed that the incidence of cardiovascular system events in patients over 75 years of age was 29.8% after adalimumab and 19 patients needed treatment (22). This study examined the reports of deaths one by one and found that the deaths were primarily in patients over 75, suggesting that clinical monitoring of the heart in elderly patients in the treatment of biopreparation RA, is necessary to avoid adverse cardiac events. Adalimumab and etanercept declined between 2013 and 2015, which was potentially related to the FDA revised reports of lymphoma in the instructions in 2013. It is possible that patients thought to have an increased risk by their physicians were then disproportionately not treated with a biological agent, thus, resulting in a subsequently lower rate of adverse events. Therefore, it is important to recognize the connection between the biological agents and regimens with cardiotoxicity events and clinical features.

This study has certain limitations in using the reports of spontaneous adverse events to produce signals. First, the nature of the voluntary reporting system may lead to reporting bias as there are still multiple factors that may affect the reported events such as lack of reporting data, incorrect event, result classifications, and misspelling of the drug name. To avoid the effects of reporting bias, we used multiple methods to encode and analyze the data. However, we cannot exclude cardiac toxicity reporting of different drugs or treatment options from being affected differently. This may result in the bias of our analysis.

Second, due to the lack of more detailed information in the FAERS database, we are unable to obtain unmeasured confounding factors that may affect the cardiotoxicity risk, such as comorbidity or pre-existing cardiovascular disease. Similarly, we could not compensate for dose modifications over time. Various forms of heart disease occur in patients with RA, such as HF and other conditions, including pericardial and myocardial disease, coronary artery disease, and disturbances in heart rhythm. Some of these conditions are more common in patients with RA than in the general population and contribute to an increased risk of death in affected patients. Ischemic cardiomyopathy is the most common cause of HF due to systolic dysfunction in the general population. HF is also likely to result from an ischemic cardiomyopathy in a significant percentage of patients with RA, given that the prevalence of coronary disease is increased in patients with RA compared with the general population (23). From our study we found that 25 patients were under the age of 16 in the study. The pediatric reports were mostly collected before the year 2005. Juvenile idiopathic arthritis (JIA) is defined as arthritis of unknown etiology that affects children before the 16th birthday and persists for at least 6 weeks with other known conditions excluded since 2001. Sometimes juvenile rheumatoid arthritis was still used during that time. Severe growth retardation, osteoporosis, and MAS are reported as common complications of the disease. Severe pulmonary complications appear to be increasing. Cardiac toxicity reporting in the juvenile patients should be noticed as they often do not have obvious cardiac diseases. However, we still need more data to analyze the biologics in the pediatric population.

In addition, we could not adjust for some cardiotoxicity risk factors not recorded in FAERS, such as smoking, dyslipidemia, comorbidities, and heredity. Third, the reporting rate may change over time, and we cannot establish a sufficient causal relationship between the target treatment and cardiotoxicity. However, our study uses the ROR method, which is considered an occurrence and not affected by the generally reported undervaluation. Finally, our findings are used to generate assumptions, not testing. Our results, therefore, lead to careful interpretation, and subsequent studies need to be performed to further explore the assumptions arising from these data.

While there are specific data and methodological limitations, our analysis of the FAERS reports also provides important hints. First, these data suggest that a higher cardiotoxicity rate was reported in adalimumab. Therefore, rheumatologists should monitor cardiac toxicity in patients with RA treated with adalimumab. Second, our findings highlight factors that may increase the likelihood and severity of cardiotoxicity and should be considered in evaluating RA treatment. However, doctors should pay more attention to screening people at high risk of cardiotoxicity, especially those treated with a drug combination that includes glucocorticoids. Oral glucocorticoid use was associated with a dose-dependent increase in the risk of HF, which was seen in patients receiving at least 5 mg daily of prednisone (HR 1.54, 95% CI 1.09 to 2.19) (9). Third, since there is evidence that the likelihood and severity of cardiac toxicity depends on various factors, doctors should also consider factors that include drug categories and cumulative dose effects (24). In addition to factors associated with rheumatoid arthritis, potential factors, including age and comorbidity (e.g., cardiovascular disease), should be considered.

Therefore, our research may help to raise awareness of the importance of interdisciplinary collaboration between cardiologists and rheumatologists to reduce heart risk while maintaining therapeutic benefits. Finally, our study may help raise awareness of the serious consequences of cardiotoxicity caused by targeted therapy, including death and disability.

So, we hope that the present findings will be considered in clinical decisions on RA treatment, and clinical studies will be designed to evaluate the obtained information from this study.

## Conclusions

Our analysis of the FAERS database identifies the cardiac outcome leading to cardiotoxicity in the targeted treatment regimen. We also identified a significant proportion of severe health results caused by cardiotoxic events. Further pharmacoepidemiologic analysis is required to evaluate the hypotheses generated by this study. While we know that our conclusions that we have drawn from the FAERS data are temporary, our findings support the ongoing monitoring and investigation of the cardiac events while using biological agents to treat RA. The results of this signal of disproportionate reporting, the first analysis studying the association between biological agents and cardiotoxicity, showed a more significant proportion of adalimumab and abatacept users linked to cardiotoxicity compared with other drugs. More research is needed in the future to explain the mechanisms that cause these phenomena and deserves to be confirmed by future clinical studies.

## Data Availability Statement

The datasets analysed during the current study are available in the Dataverse repository: https://www.fda.gov/Drugs/GuidanceComplianceRegulatoryInformation/Surveillance/AdverseDrugEffects/ucm070093.htm.

## Author Contributions

JL contributed to the conception of the study. XT and XX contributed significantly to the research, the data analysis, and the manuscript preparation, were responsible for the major part of the research. XT performed the data analyses and wrote the manuscript. BZ helped perform the analysis with constructive discussions. All authors contributed to the article and approved the submitted version.

## Conflict of Interest

The authors declare that the research was conducted in the absence of any commercial or financial relationships that could be construed as a potential conflict of interest.

## Publisher's Note

All claims expressed in this article are solely those of the authors and do not necessarily represent those of their affiliated organizations, or those of the publisher, the editors and the reviewers. Any product that may be evaluated in this article, or claim that may be made by its manufacturer, is not guaranteed or endorsed by the publisher.
